# Understanding the structural biology of osteomalacia through multiscale 3D X-ray and electron tomographic imaging: a review of X-linked hypophosphatemia, the *Hyp* mouse model, and imaging methods

**DOI:** 10.1093/jbmrpl/ziae176

**Published:** 2024-12-30

**Authors:** Daniel J Buss, Joseph Deering, Natalie Reznikov, Marc D McKee

**Affiliations:** Department of Anatomy and Cell Biology, Faculty of Medicine and Health Sciences, School of Biomedical Sciences, McGill University, Montreal, QC H3A 0C7, Canada; Faculty of Dental Medicine and Oral Health Sciences, McGill University, Montreal, QC H3A 0C7, Canada; Department of Anatomy and Cell Biology, Faculty of Medicine and Health Sciences, School of Biomedical Sciences, McGill University, Montreal, QC H3A 0C7, Canada; Faculty of Dental Medicine and Oral Health Sciences, McGill University, Montreal, QC H3A 0C7, Canada; Department of Bioengineering, Faculty of Engineering, McGill University, Montreal, QC H3A 0E9, Canada; Department of Anatomy and Cell Biology, Faculty of Medicine and Health Sciences, School of Biomedical Sciences, McGill University, Montreal, QC H3A 0C7, Canada; Faculty of Dental Medicine and Oral Health Sciences, McGill University, Montreal, QC H3A 0C7, Canada

**Keywords:** multiscale 3D imaging, EM, FIB-SEM tomography, osteomalacia, X-linked hypophosphatemia, biomineralization, bone, enthesis fibrocartilage, crossfibrillar mineral tessellation, stenciling principle

## Abstract

Biomineralization in bones and teeth is a highly regulated extracellular event. In the skeleton, mineralization at the tissue level is controlled within the collagenous extracellular matrix by both circulating and local factors. While systemic regulation of mineral ion homeostasis has been well-studied over many decades, much less is known about the regulation of mineralization at the local level directly within the extracellular matrix. Some local regulators have been identified, such as tissue-nonspecific alkaline phosphatase (TNAP), phosphate-regulating endopeptidase homolog X-linked (PHEX), pyrophosphate, and osteopontin, and others are currently under investigation. Dysregulation of the actions of enzyme-inhibitor substrate pairs engaged in mineralization (as we describe by the *Stenciling Principle* for extracellular matrix mineralization) leads to osteomalacic “soft bone” diseases, such as hypophosphatasia (HPP) and X-linked hypophosphatemia (XLH). This review addresses how advances in 3D imaging tools and software now allow contextual and correlative viewing and interpretation of mineralized tissue structure across most length scales. Contextualized and integrated 3D multiscale data obtained from these imaging modalities have afforded an unprecedented structural biology view of bone from the macroscale to the nanoscale. Such correlated volume imaging data is highly quantitative, providing not only an integrated view of the skeleton in health, but also a means to observe alterations that occur in disease. In the context of the many hierarchical levels of skeletal organization, here we summarize structural features of bone over multiple length scales, with a focus on nano- and microscale features as viewed by X-ray and electron tomography imaging methods (submicron μCT and FIB-SEM). We additionally summarize structural changes observed after dysregulation of the mineralization pathway, focusing here on the *Hyp* mouse model for XLH. More specifically, we summarize how mineral patterns/packs at the microscale (3D crossfibrillar mineral tessellation), and how this is defective in *Hyp* mouse bone and *Hyp* enthesis fibrocartilage.

## Graphical Abstract

**Graphical Abstract:**
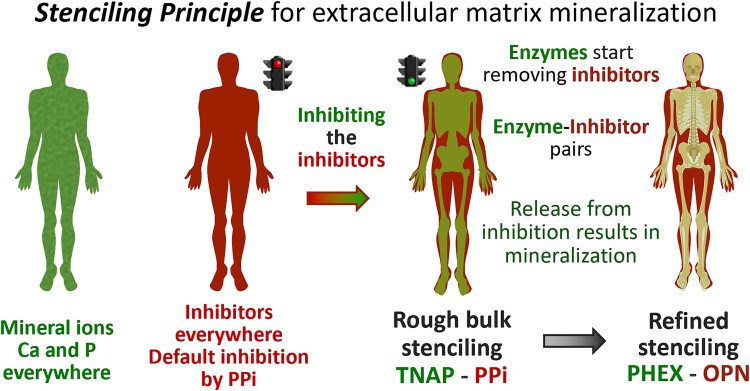

## Introduction

Skeletal pathologies, diseases, and defects can have many underlying causes. Excluding trauma, cancer, and infections (among others), one can roughly categorize skeletal diseases into “common” and “rare” in terms of incidence in the population. Common diseases, such as osteoporosis or osteoarthritis, are often multifactorial, where different pathophysiologic processes result in a characteristic spectrum of clinical manifestation. Rare monogenic diseases can affect the skeleton, and often the dentition, such as in hypophosphatasia (HPP) and X-linked hypophosphatemia (XLH). They originate from single-gene mutations that produce peculiar salient symptoms, such as unexpected spontaneous bone fractures or pseudofractures, extreme variation in stature and proportions, unusual flexibility, and/or pronounced deformities of bones.

In many cases of skeletal and dental diseases, the underlying pathobiology results in alterations of basic molecular and structural relationships between the organic and inorganic constituents of bones and teeth such that mineralization is defective.^1–5^ This is most obvious in the osteomalacic (and odontomalacic) diseases, such as HPP and XLH, where defective organic-inorganic relationships at small length scales (nano and micro) lead to clinical presentations at larger length scales in bones and teeth. To understand the consequences of disease progression arising from altered molecular interactions in mineralized tissues, and to rationally derive appropriate therapeutic strategies, the nanoscale and microscale origins and features of disease must also be understood. Recent developments in X-ray and EM imaging now allow quantitative structural assessment in 3D across all scales. In this review, we summarize our recent multiscale and multidimensional investigations that have been performed on the osteomalacic *Hyp* mouse model of XLH as an example of how we study the structural biology of a mineralization disease. We highlight how 3D multiscale imaging methods have contributed to an enhanced understanding of the mineralization defects in this disorder.

There are three particular recent advances that have significantly increased our mechanistic understanding of XLH at the structural biology level: (1) the development and wider use of advanced X-ray-based 3D imaging techniques particularly suitable for mineralized tissues, including more versatile CT methods for intermediate-scale 3D quantitative comparative analyses, (2) focused ion beam–scanning electron microscope tomography (FIB-SEM) that now provides for high-resolution (nano- and microscale) 3D quantitative analyses of tissue volumes, and (3) the advent of high-throughput 3D image-processing algorithms for unbiased analysis of large datasets and intuitive and approachable data visualization through 2D and 3D graphic renditions.^6–9^ Here, we review how we have used these three advances to achieve a better understanding of XLH. Also in this review, we broadly summarize a contemporary understanding of a double-negative (inhibiting the inhibitors) regulatory mechanism for controlling mineralization at the organismal level—a notion that we have called the *Stenciling Principle* for extracellular matrix mineralization.^10^ In the context of XLH, the principle provides mechanistic insight into its pathology. At a broader level, the principle may have implications to other cases where mineralization is adversely affected, including circumstances of ectopic calcification in soft tissues.

## The hierarchical organization of bone in 3D

Understanding Nature’s “design” of bone requires an appreciation of its hierarchical structure. Being hierarchical means that structural assemblies form nested, repeating structural units, which in turn form a higher-level hierarchical unit, and so on.^11^^,^^12^ This feature of hierarchy applies to both the organic and inorganic phases of bone. At no size scale is bone a homogeneous “material”—it is always a “structure”—and thus it is heterogeneous.^13^^,^^14^ Such hierarchies can also be self-similar or fractal-like in nature, as is the case for bone (currently documented are 12 hierarchical levels).^15^ Hierarchical organization is central to the remarkable mechanical properties of bone and its performance in a properly functioning skeleton.^12^^,^^16^^,^^17^ From this, one might readily surmise that a breakdown in this hierarchy, at any level, might then form a basis for skeletal pathology and disease with clinical manifestations. Thus, it becomes imperative to carefully document and understand the hierarchical organization of normally functioning archetypal bone. As to be expected, variations among species most certainly exist, such that the hierarchical organization of bone in a short-lived mouse (for example) would be “abridged” in comparison with human bone, while the bone of ungulates displays remarkably even higher complexity than human bone. Yet, despite species-specific differences at the tissue level, the basic submicrometer modules of bone structure are comparable between small and large animals,^18^ and generalizations can indeed be made, as we discuss here for mouse and human.

Accordingly, the structure of bone must be interrogated across the multiple size scales where structural details can be placed into their broader context. Use of multimodal imaging must be implemented in a correlative manner to capture the specific context of multiscale structural complexity. For hierarchical structures, context is as important as resolution in addressing local inhomogeneity, growth and development trajectories, and for finding mechanistic links between structural defects and clinical manifestations. The choice for using either a contextual or a high-resolution technique depends on the question being asked. For the study of rare mineralization diseases as discussed here, the combined use of micro-CT (μCT, X-ray microscopy) and FIB-SEM tomography is sufficient to span 5-6 orders of magnitude in scale. Such a scale allows for 3D quantitative observations of (1) morphology of the skeleton and bones, (2) cells such as osteocytes and their dendritic cell processes,^19^^,^^20^ and their interfaces with the mineralized extracellular matrix in the lacuno-canalicular network (LCN),^21–23^ (3) collagen fibril organization in the osteoid and mineralized bone,^11^^,^^23–25^ and (4) nanoscale mineral crystallites and their aggregate packing at the microscale to form the 3D crossfibrillar tessellation pattern characteristic of lamellar bone.^23^^,^^26^^,^^27^

## The *Stenciling Principle*: regulation of mineral patterning in bone

In health, diet and homeostatic hormonal regulation of calcium (Ca) and phosphate [PO_4_]^3−^ ions provide a continuous and plentiful source of circulating mineral ions for metabolic/physiologic needs. A continuum of generally equilibrated tissue fluid composition throughout the body well beyond the circulation provides an essential pathway for mineral ions to traverse connective tissues.^28^ Importantly, in physical chemistry terms, and in the sense of mineral ion concentrations and the driving forces required to precipitate Ca-P mineral (in this case being highly supersaturated with respect to the mineral phase of bone, carbonate-substituted hydroxyapatite), mineral should in principle easily and spontaneously precipitate in essentially all connective tissues^29^^,^^30^—where in fact it does not—the mineral ions essentially stay instead in metastable equilibrium. Why does a mineral not normally precipitate spontaneously in the collagenous dermis of the skin, or the extracellular matrix-rich vascular wall?

**Figure 1:**
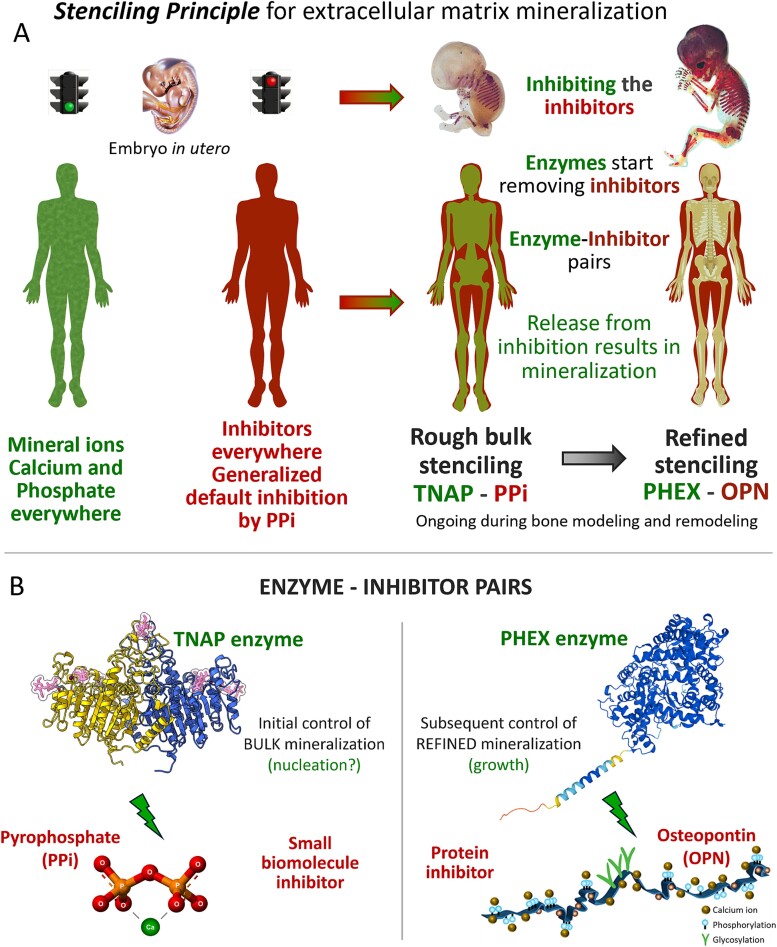
The *Stenciling Principle* for extracellular matrix mineralization builds upon concepts initially presented by F. Jacob and J. Monod for the double-negative (repressing a repressor) genetic regulation of enzyme expression in the *lac operon* in bacteria, and then to some degree by W. Neuman, H. Fleisch and G. Russell to describe pyrophosphate metabolism and alkaline phosphatase enzyme effects on crystal growth (inhibiting the inhibitors). The *Stenciling Principle* postulates the following: (A) In body fluids normally abundant in calcium and phosphate ions, mineralization is largely inhibited by ubiquitous pyrophosphate (PPi), a potent inhibitor of mineralization. Abundant expression of TNAP by cells in skeletal tissues to cleave inhibitory PPi at the site of future mineralized tissue releases the inhibition, and mineralization begins. At the protein level, SIBLING family member osteopontin (OPN) is highly negatively charged and highly phosphorylated, and it binds ionic and mineral calcium to inhibit (slow, regulate) mineralization. Cell-specific expression of phosphate-regulating endopeptidase homolog X-linked (*PHEX*) exhaustively degrades OPN into small, noninhibitory fragments. This second level of finely tuned, perhaps more sustained protein degradation may ensure timely growth of mineral crystallites, and refinement of mineralization patterning at cell-matrix interfaces such as in the LCN and at the mineralization front (where OPN is abundant). (B) Schematic showing the enzyme-inhibitor/substrate pairs that form the basis of the *Stenciling Principle* as described above. TNAP structure from,^40^ and PHEX structure from AlphaFold.

The *Stenciling Principle* we have proposed^10^ invokes a global default inhibition state actuated by the small biomolecule inorganic pyrophosphate (PPi) for mineralization inhibition in connective tissues.^28^^,^^31^ Tissue-specific release from this global inhibition (“stenciling”) occurs through the enzyme-producing actions of resident cells specifically in bones and teeth to degrade PPi, providing a double-negative (inhibiting the inhibitor) regulation of initial extracellular matrix mineralization that allows it to proceed. The outcome of this initial enzyme-inhibitor pair reaction (TNAP/PPi; tissue-nonspecific alkaline phosphatase/PPi) to degrade PPi inhibitor “kickstarts” the mineralization process.^30^^,^^32^^,^^33^ Then, within mineralizing tissues, this is followed by the actions of a second enzyme-inhibitor pair reaction (PHEX/OPN; phosphate-regulating endopeptidase homolog X-linked/OPN) to degrade^34^ the inhibitory protein osteopontin^35–38^ more slowly throughout this regulatory process for mineralization. The combination of general and fast release-from-inhibition by the PPi/TNAP pair (“crude” stenciling), followed by local and slower release-from-inhibition by the OPN/PHEX pair (“fine” stenciling), ensures an initial robust mineralization initiation within the bone matrix, and its physiologic progression for fine-tuning of stiffness and toughness in bone.^10^^,^^18^ In the context of the osteomalacic mineralization diseases, consistent with this *Stenciling Principle* are the observations that in patients and mouse models with inactivating mutations in the *TNAP* (*TNSALP*, *ALPL*) gene there is an accumulation of mineralization-inhibiting PPi in the bone matrix causing HPP.^32^^,^^39^ With inactivating mutations in the *PHEX* gene, there is an accumulation of mineralization-inhibiting OPN in the bone matrix, causing XLH.^1^^,^^33–35^  Figure 1 summarizes the notion of the *Stenciling Principle*.

## Symmetry breaking, nascent mineral foci patterning, and mineral tessellation

Natural structures and assemblies are characterized by recognizable patterns, and spontaneous symmetry breaking is the origin of most patterns. Symmetry breaking is widely studied in many different fields, including physics, chemistry, and biology. Contrary to the colloquial notion of symmetry (eg, mirror symmetry), a perfectly monotonous and featureless structure is considered to possess the highest possible degree of symmetry^41^ Biological equilibria are often metastable, and random fluctuations of local “order” disrupt uniformity and can generate a pattern simply by exceeding a local threshold switch, a classic example being reaction–diffusion systems that often produce stunning and universal patterns in Nature.^42^ Dynamic biological systems continuously break featureless symmetry, which in turn generates new constraints, patterns, and equilibria. Biomineral patterning seems to be no exception, and a common motif indeed is a surface-filling, or volume-filling tessellation in mineralized tissue structure at different scales, as exemplified by mineralized shark and ray cartilage tesserae, macroscale tessellated turtle carapace bones, and intercalated mollusk nacre.^43–47^

In bone, the collagenous matrix of the osteoid forms initially as a relatively “featureless” organic canvas, where mineralization soon ensues, attributable to the metastable equilibrium of mineralization promoters and inhibitors.^10^^,^^48^ In lamellar bone (the most common bone type in a mature mammalian skeleton), incipient mineralization foci form with a quasi-periodic spacing within the collagen-rich osteoid and near the mineralization front to swiftly grow through Voronoi-like patterning into a 3D microscale space-filling assembly, which we have called 3D crossfibrillar mineral tessellation.^49–51^ Closely packed individual mineral tesselles (geometrically approximating prolate ellipsoids) abut against each other while maintaining narrow interfaces that persist throughout the structure of mature lamellar bone.^18^^,^^23^^,^^26^ When symmetry breaking is easy to trigger (low threshold), mineralization foci form as a dense population and grow into small and uniform tesselles, separated by a collectively large area of interfaces. This happens in healthy bone. For example, TNAP enzyme expression by osteoblasts and osteocytes to lower PPi inhibition of mineralization is likely is a mechanism that creates a low-threshold trigger for mineralization foci formation at the nanoscale—a heterogeneous nucleation event where multiple and similar motifs appear simultaneously and independently. Once heteronucleation takes place, subsequent growth and volume-filling tessellation of the mineral itself is governed by the equilibrium of promoters/inhibitors.^18^^,^^30^^,^^48^ Subsequent PHEX cleavage of mineralization-inhibiting OPN protein^34^^,^^52^ unleashes the growth of the foci toward the tessellation of mineral into a volume-filling 3D assembly as is found in bone. However, even when symmetry breaking takes place as it does in XLH/*Hyp* bone (see below), where abundant mineral foci appear in the osteoid similar to healthy bone, the loss of PHEX enzymatic activity in XLH/*Hyp* subsequently cannot remove lingering inhibitory OPN.^35^^,^^52–55^ The mineralization foci do appear, but they essentially fail to grow into a tessellated volume-filling assembly of uniform tesselles.^23^^,^^49^ From this, the mineral content and periodicity/regularity of the resultant pattern are decreased, and this has dire consequences in terms of mechanical competency. In all cases, the texture of the collagenous matrix defines the anisotropy of the tesselles.^23^^,^^24^

## Details of 3D crossfibrillar mineral tessellation in healthy WT mouse bone

**Figure 2:**
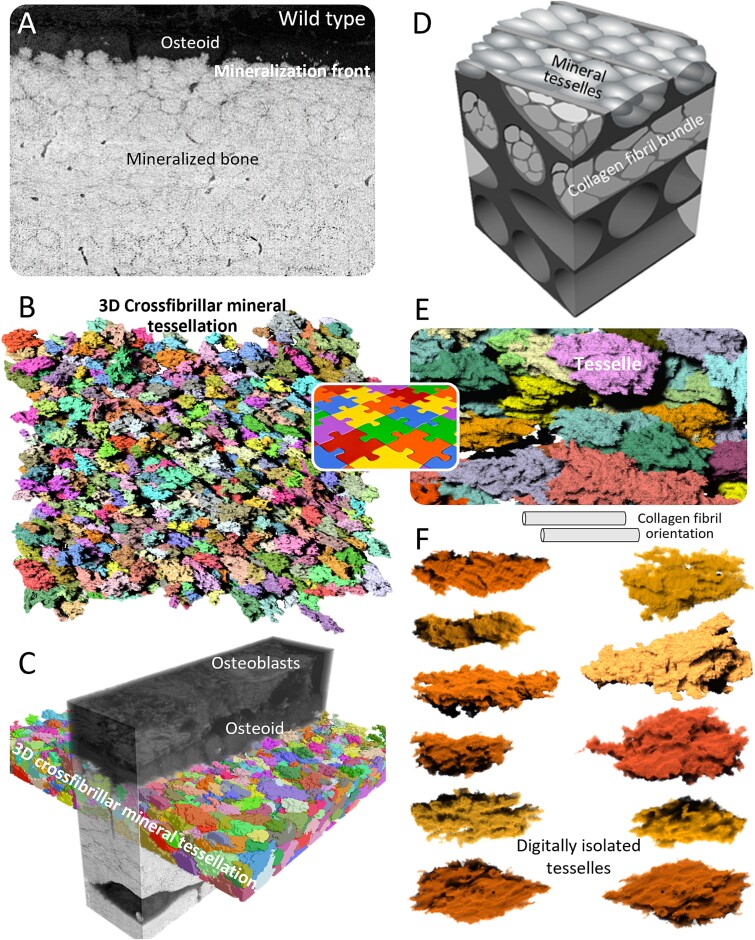
Healthy WT mouse lamellar bone and 3D crossfibrillar mineral tessellation as assessed by FIB-SEM tomography. (A) A 2D image from a 3D FIB-SEM volume, oriented across the mineralization front. A “cobblestone” mineral packing pattern with discrete, discernable darker boundaries is noticeable. (B) Deep learning-aided segmentation of mineral and application of a watershed transform labels individual abutting/tessellating mineral volumes (mineral tesselles) geometrically approximating prolate ellipsoids. (C) 3D visualization of greyscale data showing watershed segmentation of tesselles in the first two bone lamellae beyond the osteoid. (D) Schematic representation of data from various FIB-SEM volumes describing how the long axis of mineral tesselles follows the directionality of the collagen fibril bundles in alternating lamellae, with 3-4 tesselles incorporated across each collagen bundle cross-section. Collagen bundles change direction in each lamella, which the directionality of the mineral tesselles follow. (E) From a high-resolution 3D FIB-SEM volume, a rendering of closely packed, individual mineral tesselles near the mineralization front. (F) Individual mineral tesselles digitally isolated from the full 3D volume display some variation in size and shape but generally approximate a prolate ellipsoid geometric shape. Figure adapted from references 18 and 23.

At the scale of microns, healthy mature lamellar bone is characterized by 3D assemblies of mineralized collagen fibrils grouped into gently twisting bundles, and organized as ordered arrays having alternating directions.^24^ In lamellar bone formation, the collagenous osteoid assembles first, and then mineralizes at the so-called mineralization front (Figure 2A), while another array of collagen bundles is deposited behind it by osteoblasts.^49^^,^^51^ It is within this pre-assembled, collagenous extracellular matrix in the osteoid that the formation of microscale units called tesselles begin—as small mineral foci.^50^^,^^51^ The foci are the “seeds” of future tesselles,^22^^,^^49^ that ultimately grow and approximate their nearest neighbors as they expand in all directions at the microscale as enlarging mineral aggregates (Figure 2B and C). Their growth is not equal in all dimensions, but rather occurs preferentially along the long access of collagen fibrils. In WT mouse lamellar bone, average dimensions for a tesselle are 1.6 × 0.8 × 0.8 μm with a length range varying from 1.5 to 2.5 μm, and their average volume is ~1 μm^3^.^18^ With an aspect ratio of 2, they have an elongated shape geometrically approximating a prolate ellipsoid.^23^^,^^26^^,^^56^ Multiple tesselles span a single ~2 μm bundle of collagen fibrils (Figure 2D), and thus multiple tesselles pack within a thickness of one lamella.^23^ At the mineralization front, which can be viewed as a snapshot of bone formation and mineralization, the distance between a most recent mineral focus in the osteoid and the fully-formed, volume-filling tessellation occurring at the mineralization front rarely exceeds 2 μm, meaning that the time elapsing between foci heteronucleation events and mineral tessellation is <1 day^57^—a truly precipitous process. In the *Hyp* mouse, however, the distance of the isolated (aborted, severely impeded, or temporarily arrested) nonspace-filling tesselles from the mineralization front exceeds tens of micrometers,^23^^,^^49^ illustrating that after nucleation takes place, the growth process of the mineral tesselles is slowed down by at least an order of magnitude.

At the length scale needed to visualize and quantify such microscale tesselle structures and how they relate to one another as they pack into a volume-filling assembly, focused ion beam–scanning electron microscopy (FIB-SEM) tomographic imaging (serial-surface-view, also known as slice-and-view, see Addendum) was used to understand the details of this mineral pattern in 3D (Figure 2E). Quantitative metrics can be extracted from the analyzed volume when coupled with deep learning-assisted feature segmentation and application of a watershed transform.^6^ Our original analysis quantified features of over 5000 individually labeled (segmented) tesselles in a single FIB-SEM volume of mouse tibial lamellar bone.^23^ Quantitative analysis by thickness mapping in 3D across a volume of bone tissue highlights the statistical power of comparing thousands of observations. Without a semi-automated, high-throughput 3D image analysis workflow, only descriptive 2D observations of such a mineral pattern were previously available. From this updated body of work, crossfibrillar 3D mineral tessellation is now considered to be a ubiquitous feature of lamellar bone,^18^^,^^23^^,^^26^^,^^27^ enthesis fibrocartilage, and tendon (calcifying tendinopathy),^49^ with some heterogeneity occurring across tissues and species.^10^ The important mechanical implications of such a repetitive patterning/tessellation rich in organic-inorganic interfaces^23^^,^^58^^,^^59^ are that while mineralization confers stiffness to the extracellular matrix, the numerous interfaces contribute to its toughness.^60^^,^^61^  Figure 2F highlights features of digitally isolated tesselles viewed from different angles and rotations.

## Physiologic bone mineralization versus pathologic osteomalacia (hypomineralization)

Most biological processes balance a trade-off of conflicting properties, requiring not too much, not too little, but something just right. Mineralization is no exception. Within a narrow range, higher mineral content is useful to resist deformation, as in a rigid frame of a bird wing or in the dense middle ear ossicles. Again, within a narrow range, slightly less mineral content renders bone resilient, which is beneficial for juvenile skeletons, where young animals of all species tend to “fall off, from and out of things”.^62^ “Stepping out” of the physiologic lower boundary of the mineralization range is characterized by soft bones that deform too easily. Indeed, hypomineralization occurring either through vitamin D-deficiency rickets, or through hereditary vitamin D-resistant rickets (XLH), shares very similar soft bone deformation manifestations.^63^ The most abundant mineral phase of bone—a carbonate-substituted hydroxyapatite—constitutes an estimated 65%-70% of its weight,^64^ which is the physiologic range, as regulated by the *Stenciling Principle.*^10^ In addition to controlling the extent of bulk collagenous matrix mineralization, the PHEX/OPN pair maintains an appropriate mineral content at the interface with bone-embedded osteocytes.^1^^,^^34^^,^^36^

## XLH and the *Hyp* mouse model: a mechanistic and structural perspective

From a structural perspective, in 1973, Steendijk and Boyde remarked that “The mineralizing fronts of the bone from patients with hypophosphataemic rickets typically showed an unusually wide range of orientation of the unjoined mineral particle clusters compared with normal bone”.^65^ Despite not knowing the origins of this structural observation at this time, seminal work using electron microscopy offered a first glimpse into the accumulation of local inhibitors in the extracellular matrix of bone. From a mechanistic perspective, since the identification of the phosphate-regulating endopeptidase homolog X-linked gene (*PHEX*/*Phex*),^66^ and the subsequent discovery that mutations in this gene cause XLH,^67^^,^^68^ much has been learned about both the systemic and local effects of PHEX enzyme inactivation. PHEX is highly expressed in healthy mineralized tissue cells, such as osteocytes, osteoblasts, and tooth odontoblasts, with much lower levels present in some other tissues.^49^^,^^67^^,^^69^ Spontaneous truncating mutations identified in mice led to the discovery and widespread use of the *Hyp* (*Phex^Hyp^*) mouse, which phenocopies XLH in humans (In humans, inactivating mutations in the *PHEX* gene decrease the enzymatic activity of PHEX, resulting in dramatic elevations in FGF23, a critical hormone regulating phosphate homeostasis in the kidney.^70^ FGF23 reduces renal phosphate reabsorption through its effects on the sodium/phosphate cotransporter NaP_i_-2a and 2c, and acts as an endocrine regulator of 1,25-dihydroxyvitamin D (1,25(OH)_2_D). Upon binding to FGFR1c in the presence of α-KLOTHO, FGF23 downregulates CYP27B1 and upregulates CYP24A1, leading to decreased levels of active 1,25(OH)_2_D, and reduced intestinal absorption of phosphate.^71^ The resulting elevated FGF23 and low-to-normal levels of 1,25(OH)2D cause phosphate wasting, rickets, and osteomalacia, typically leading to soft and undermineralized bones and tooth dentin.^1^^,^^72^^,^^73^ While the exact relationship between *PHEX* mutations and elevated FGF23 remains unclear, the enzyme’s catalytic site is known for its strong specificity for aspartic acid (Asp) and glutamic acid (Glu) residues.^34^^,^^74^).^68^^,^^75^

Our studies investigating possible physiologically relevant targets for PHEX’s catalytic activity revealed its specificity for direct cleavage of full-length osteopontin (OPN) substrate and its peptides at dozens of cleavage sites.^34^ This unusual broad degradation profile of inhibitory OPN and its peptides by PHEX, resulting in small inactive fragments, is considered to be a mineralization inhibitor degradation cascade, one that gradually inactivates OPN’s ability to inhibit mineralization. Consistent with these findings, we also showed elevated OPN and OPN peptides in the *Hyp* mouse lacking PHEX.^23^^,^^73^ Our studies on human bone from XLH patients likewise showed an elevated abundance of OPN in bone and tooth dentin,^1^ particularly at the *lamina limitans* surrounding osteocyte lacunae and canaliculi, and at the mineralization front, where OPN binds directly to the crystals in forming mineral foci.^36^^,^^37^^,^^53^^,^^76^^,^^77^ Attributable to its high negative charge, enhanced by extensive phosphorylation (phosphoserine residues), and intrinsic disorder,^78^^,^^79^ the accumulation of OPN (in the absence of PHEX catalytic activity^1^^,^^34^) at these interfaces has been implicated as a contributor to insufficient mineralization. 3D collagen packing appears the same,^23^ and symmetry breaking takes place in both mouse strains, as can be seen from the similar morphology and density of the incipient foci, but then the growth of the tesselles is either aborted, severely impeded, or temporarily arrested. Large gaps remain with no interlocking and no space-filling, so that compliant behavior dominates these regions of the bone upon loading.^18^^,^^23^^,^^49^

The most significant changes occurring in the tissue-level structure of *Hyp* bone compared to healthy WT bone are (1) loss of a clearly defined mineralization front coupled with defective and incomplete mineral tessellation,^23^^,^^65^^,^^73^ and (2) peri-osteocytic lesions^1^^,^^80^ (POLs, also called hypomineralized halos; see section below). Both these changes result from defective (hypo) mineralization, and accumulation of mineralization inhibitors in the extracellular matrix locally (OPN, in the absence of its inactivator enzyme PHEX), to which FGF23-driven inadequate circulating phosphate levels contribute systemically. We conclude this section by further reviewing these mineralization deficits in *Hyp* bone compared to normal WT bone, from the nano- to the microscale, and propose roles for altered mechanical properties and cellular function.

### Defective mineral tessellation in *Hyp* mouse bone

**Figure 3:**
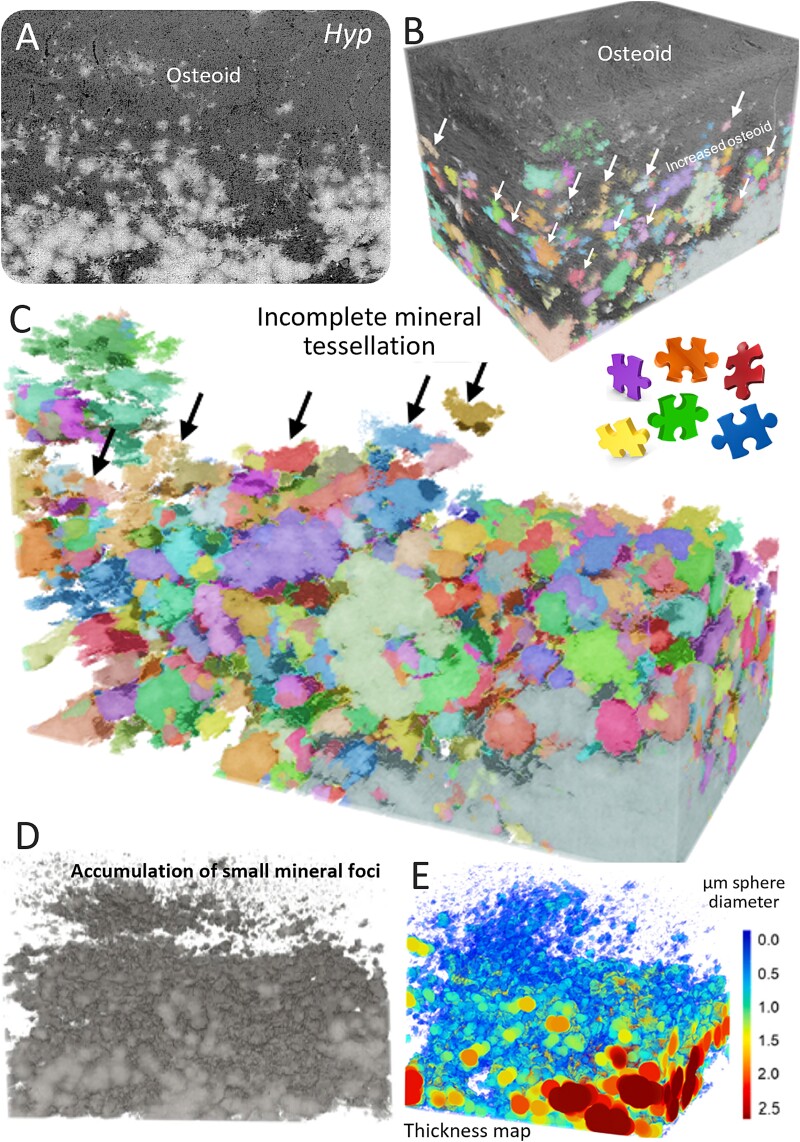
Osteomalacic *Hyp* mouse lamellar bone assessed by FIB-SEM tomography. (A) 2D image from a 3D FIB-SEM volume oriented across the mineralization front showing an incongruent and delayed trajectory of mineral packing, also observed in 3D after watershed labeling with (B) and without unmineralized collagen which has been digitally removed (C). Incomplete, nonspace-filling mineral tessellation is readily apparent. From deep learning-based segmentation in (D), thickness maps were computed in (E) to quantify the packing of mineral volumes at the mineralization front, which was used in comparison to healthy bone from WT mice. Arrows indicate individual foci/tesselles. Figure adapted from references 18 and 23.

By examining closely the morphological landscape of healthy osteoid mineralization, it is possible to compare equivalent sites at the osteoid/mineralization front of *Hyp* lamellar bone. At the diffuse, extended, and irregular mineralization front of *Hyp* lamellar bone associated with pervasive adjacent osteoidosis, there is a delay in mineral growth, rather than in nucleation.^23^ FIB-SEM tomographic 3D volumetric data revealed the prevalence of an extraordinary large number of small mineral foci in *Hyp* mice, remaining isolated, and failing to form a distinct, repetitive volume-filling mineralization pattern (Figure 3A-D). Where some degree of mineral tessellation is observed, it is incomplete and irregular.^23^^,^^73^ Using the thickness mapping feature of the reconstruction software (Dragonfly) to quantify changes in the size of mineral volumes in 3D (including of tesselles) (Figure 3E), the average mineral tesselle thickness was found to be 0.9 μm in the WT bone at the mineralization front and less than half of that at 0.4 μm at an equivalent site of circumferential lamellar *Hyp* bone (where they approached the nanometer range). Although the mineralization front is indistinct in *Hyp* mice compared to WT mice, an approximate expanded region of mineralization can nevertheless be identified and its relative location to the nearby osteoblast layer determined. Accordingly, all foci/tesselles included within the identified and analyzed volumes were measured, and both WT and *Hyp* tissue volumes were chosen to be approximately the same size.

**Figure 4:**
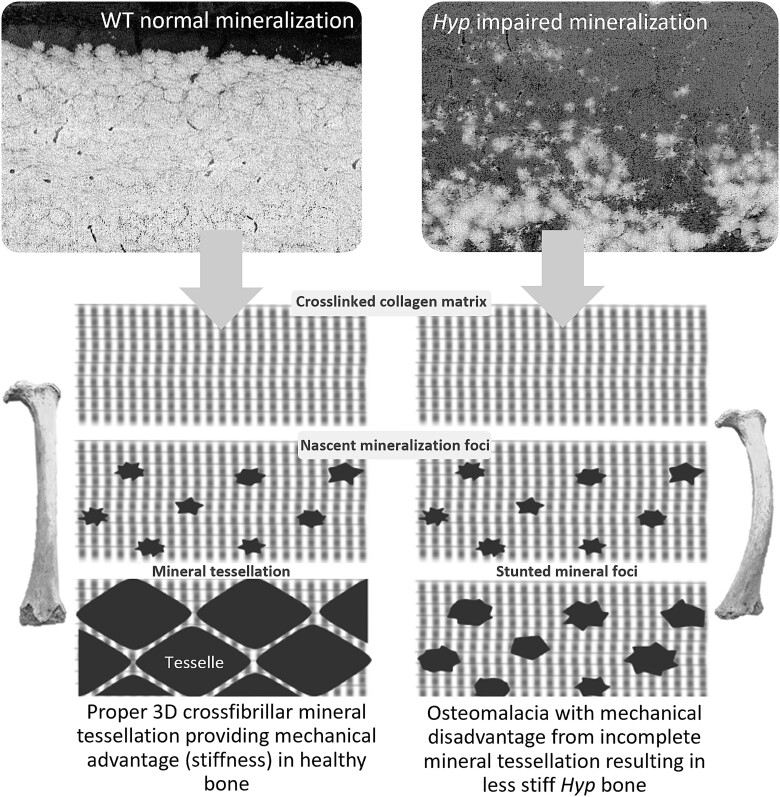
Comparison of close-packed crossfibrillar mineral tessellation in healthy WT mouse lamellar bone versus delayed and incomplete mineral tessellation in *Hyp* bone. In normal WT bone small mineral foci in the osteoid grow into larger roughly prolate ellipsoid-shaped mineral tesselles conferring stiffness and compression resistance at interfaces under loading. In osteomalacic *Hyp* mouse lamellar bone, an impaired mineralization trajectory and sparse/irregular packing of mineral tesselle volumes results in less interlocking and higher flexibility, which enables macroscale bone deformities such as bowed legs, knock-knees and buckling pseudofractures. The top micrographs show in 2D differing mineralization patterns occurring across the mineralization front. The diagrams under the micrographs roughly illustrate the progression from unmineralized osteoid, to nascent mineralization (symmetry breaking), to space-filling tessellation in WT, or lack thereof in *Hyp*. Figure adapted from reference 23.

Such structural observations revealing delayed and incomplete tessellation of mineral units in the *Hyp* mouse clearly would alter its mechanical properties in compression and bending, leading to bone deformation and buckling (pseudofractures) over time, features that are also hallmarks of the human disease.^81^ The anticipated mechanical behavior of *Hyp* bone that has delayed mineral foci growth and smaller tesselles would reduce bulk stiffness resulting in characteristic long bone deformities (eg, *genu valgum* or *genu varum*).^23^^,^^45^^,^^60^ Mineralization deficiencies at the microscale and nanoscale are not resolvable on radiographs and on most 2D sections stained with von Kossa/Toluidine Blue or Goldner’s Trichrome. Other methods like SEM-BSE imaging of the blockface do not capture the full volumetric context and, importantly, restrict the accuracy of quantification depending on the plane of section. Observation of mineral tessellation is also not always trivial because it requires an ultra-smooth surface and careful selection of imaging conditions to resolve fine nanointerfaces. An abnormal and more heterogeneous defective 3D crossfibrillar mineral tessellation likely contributes to altered global mechanical properties in XLH/*Hyp*, the most important being changes in stiffness that would be mechanically disadvantageous^82^ (Figure 4). Indeed, reduced quantity and quality of mineral in *Hyp* mice has been linked to specific bone mechanical deficits, namely a significant increase in femoral angular deformation at failure, occurring with no significant change in polar moment of inertia (which could otherwise impact torsional behavior).^83^

### POLs in *Hyp*/XLH

**Figure 5:**
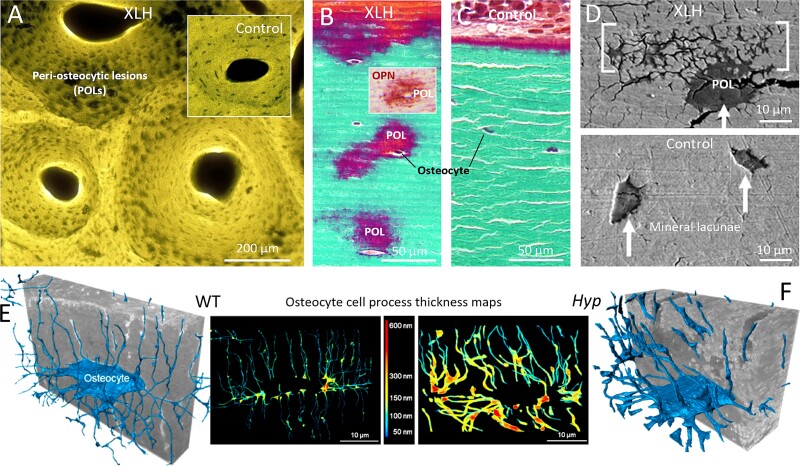
Peri-osteocytic lesions (POLs) and osteocyte cell-matrix/mineral relationships. (A) Contact microradiograph of a human bone slice showing dark, unmineralized peri-osteocytic lesions “halos” in an XLH patient bone (inset of normal mineral lacunae appearance in human control bone). (B) Goldners trichrome-stained undecalcified histology section corroborating the occurrence of large peri-osteocytic, unmineralized lesions (in red; green is mineralized bone) in an XLH patient, and also showing an accumulation in POLs (by immunolabeling) of mineralization-inhibiting OPN (inset). (C) Comparison with Goldners trichrome staining of control bone illustrating the vastly enlarged osteocyte mineral lacunae in XLH. (D) Blockface backscattered SEM imaging of human XLH bone (top panel) and control bone (bottom panel) showing a magnified view of a POL and the enlarged-diameter canaliculi extending from the lacuna (brackets). Arrows indicate osteocyte mineral lacunae. (E) A segmented osteocyte from WT mouse lamellar bone with cell processes near the mineralization front (segmented using deep learning; the actual cell processes are imaged, not the canaliculi of the LCN), and thickness map, showing cell processes generally being around 100-150 nm in thickness. (F) A segmented osteocyte with cell processes from *Hyp* osteomalacic bone near the ‘mineralization front with the thickness map showing cell processes being up to 500 nm in thickness. Figure adapted from references 1 and 23.

Peri-osteocytic lesions are mineralization defects present as halos of unmineralized extracellular matrix surrounding embedded mature osteocytes in the bone of XLH patients (Figure 5A-D) and *Hyp* mice (Figure 5E and F). They are typically more pronounced than the thinner halos observed in osteocytic osteolysis. Measuring tens of thousands of osteocyte mineral lacunae in entire tibiae of WT and *Hyp* mice by 3D synchrotron μCT, we determined that the *Hyp* mean mineral lacunar volume of 595 μm^3^ was substantially much greater than the WT mean mineral lacunar volume of 405 μm^3^.^80^ These large-scale 3D findings corroborate many 2D histology findings for POLs, and additionally quantify the actual volume differential of the POLs in large volumes of mouse bone tissue. The causes of enlarged POLs are likely from both low serum phosphate and/or increased local inhibitor accumulation (perhaps OPN and/or PPi) within the lacunae. Detailed 3D imaging of *Hyp* and *Hyp*;*Opn*^−/−^ mice, in addition to recent biochemical and metabolic studies, have investigated the origins of POLs found in XLH/*Hyp* bone. Overall, these studies suggest predominant upstream roles for 1,25D and PPi, with OPN likely being even further upregulated by Pi and PPi to inhibit mineralization.^33^^,^^84^^,^^85^ While studies have shown that the previously conventional treatment of XLH patients with phosphate and vitamin D can resolve to some degree the POLs (depending upon therapy compliance),^73^ it is not yet clear how this is effected in terms of regulating inhibitors of mineralization at that very local level in the extracellular matrix. While counterintuitively inhibitory OPN would be expected to be upregulated in POLs by the vitamin D/phosphate treatment, very little is known about how the treatment affects inhibitory pyrophosphate levels and TNAP and PHEX expression, these being important players (among others) in this regulatory control of mineralization. Also, it is likely that the phosphorylation status of inhibitory matrix proteins such as OPN is important, as regulated by kinases and phosphatases, such as FAM20C and TRAP (discussed in^33^ and^72^). Finally, while it is known that mineralization is quite heterogeneous in XLH patients,^73^ why some osteocytes do not develop surrounding POLs remains to be adequately explained.

Indeed, from these findings and from the evidence for a soft pliant zone of peripheral unmineralized matrix at the osteocyte lacunar wall rich in mineralization-inhibiting OPN^1^(Figure 2B)—rather than the OPN-rich, clearly defined thin organic coating known as the *lamina limitans* found in normal bone^1^^,^^77^—we propose that the defective mineralization of the POLs is a result of local inhibitors of mineralization that occupy an upstream role in modulating the FGF23 levels responsible for renal phosphate wasting in the XLH condition. A softer and more pliant perilacunar matrix around many POL osteocytes (not all osteocytes have POLs in XLH/*Hyp*) may well have a substantial role in modifying intracellular signaling pathways, distribution of organelles, and gene/protein expression,, including secretion of hormones such as FGF23. Initial support for this idea is the observation that the actual osteocyte cell processes in three dimensions are substantially thicker in *Hyp* than in WT bone.^23^ This pronounced change in osteocyte cell morphology captured using high-resolution FIB-SEM imaging with specimen cryo-preservation (vitrification) techniques (Figure 5E and F) may hint at similarly dramatic alterations occurring within the osteocytes occupying POLs. This initial role of LCN-localized OPN is supported by recent 2D work using bone tissue sections showing that treatment with FGF23-neutralizing antibodies (burosumab) does not correct POLs.^86^ It is likely that detection of altered strain fields in the POLs (affecting osteocyte mechanosensation) is facilitated by variations in the ratio of unmineralized to mineralized bone matrix (perhaps using osteocyte CD44 or α_v_β_3_ receptors for which OPN has ligands), activating an as yet unknown pathway responsible for increasing FGF23 release by osteocytes.^87^ This is supported by the finding that mice deficient in DMP1 also have POLs and show increased FGF23 expression in osteocytes but not in osteoblasts.^80^ A critical aspect of POL progression may also be the fact that FGF23 overexpression has been linked to downregulation of TNAP, further increasing the PPi/Pi ratio that in turn also results in increased OPN,^33^^,^^85^ creating a feedback loop that exacerbates mineralization defects. These 3D data suggest that osteocyte and cell process morphologies in the LCN may change significantly, attributable to the perilacunar mineralization state in select skeletal pathologies, with POL-mediated changes in mechanosensation likely thus also interfering with signaling to cells at the bone surface. Although burosumab treatment has positive effects in addressing osteomalacia and improving overall skeletal growth and correcting deformities, it is clear that some aspects of XLH are not solely dependent on the effect of PHEX inactivation influencing mineral ion homeostasis through the systemic actions of circulating hormones. Other mechanisms are clearly at play, particularly those arising locally from osteocyte matrix/mineral interactions.^86^^,^^88^

Tokarz et al. suggest that enlarged mineral lacunae in *Hyp* mice could also be a result of an observed increase in LCN-regulating genes such as *CTSK*, *ALPL* (*TRAP*), and *MMP13* restored upon daily treatment with 1,25D or FGF23-neutralizing Ab.^89^^,^^90^ It remains unclear what the true magnitude of the structural effects at the nano- to microscales of these altered gene expression patterns is. While it could be possible that the action of these molecules contributes to POLs, given the more meager structural changes that are observed during osteocytic osteolysis when these molecules are also upregulated,^91–93^ questions remain. It has been suggested that osteocytes are capable of resorbing both mineral and the collagenous matrix. In MMP13-deficient mice, this protease is shown to be essential for bone quality, even outside of lactation-induced osteolytic osteolysis.^94^ Despite increased *MMP13* and *CTSK* expression in *Hyp* mice (suggesting a mineral and organic matrix resorption analogous to the suspected mechanism of osteocytic osteolysis), Goldner’s trichrome staining of XLH patient bone instead shows an unmineralized collagen template that remains in each POL.^1^ These findings highlight the urgent need for investment in correlative and multiscale high-resolution 3D imaging pipelines that can resolve both inorganic and organic constituents (including structural preservation with cryo-vitrification), to accompany molecular and biochemical investigation. Such imaging is essential for understanding the extent of perilacunar remodeling and structural alterations associated with normal physiology and pathologic processes in bone.

## Defective crossfibrillar mineral tessellation in *Hyp* mouse fibrocartilaginous enthesis

The function of the enthesis is to effectively transfer mechanical stress between tissues having dissimilar mechanical properties, between flexible tendon and stiff bone. Nature’s design solution to ensure fail-safe function at the tendon attachment to bone is through a precise gradient of elastic modulus. This unique stiffness gradient found across the enthesis—which remarkably spans as little as hundreds of micrometers in the mature enthesis of mice—develops as a result of complex gene expression patterns during development.^95^^,^^96^ These developmental cues result in juxtaposed zones having distinct extracellular matrix compositions and organization, and with a matching mineralization and hydration status.^97^ At the Achilles enthesis, mineralization occurs within the fibrocartilage zone where the tendon inserts, and the calcified fibrocartilage connects with the underlying calcaneal bone through a corrugated cement line/plane.^98^^,^^99^

**Figure 6:**
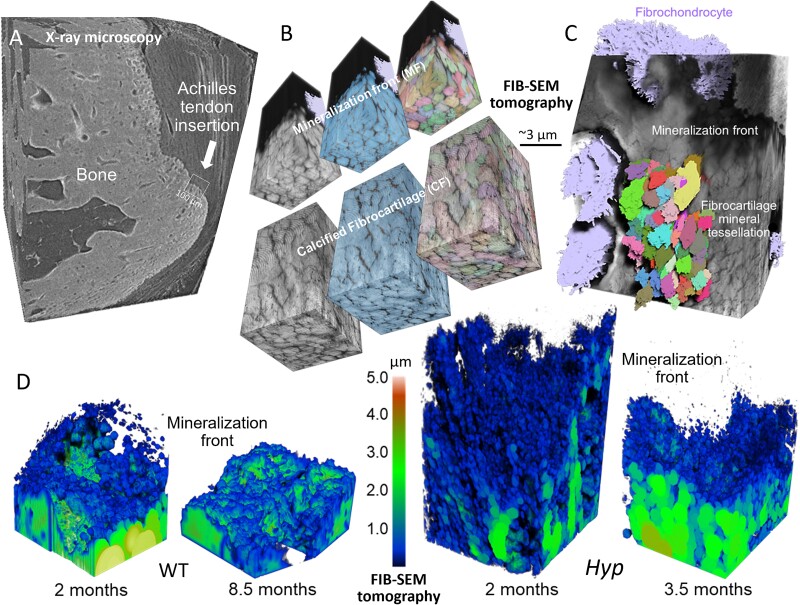
Multiscale 3D imaging of the mouse Achilles tendon fibrocartilage enthesis. (A) Submicron X-ray microscopy CT volume of normal WT mouse Achilles enthesis and underlying subchondral bone, prepared by critical-point drying. (B) Small FIB-SEM volume from a normal WT mouse at a region approximately shown in (A) showing greyscale data, deep learning-aided semantic segmentation labeling of mineral (blue) and corresponding instance or watershed segmentation for mineral tesselles (multi-colored). A fibrochondrocyte near the mineralization front is segmented and colored in purple. (C) Mineral tesselles from (B) are shown in the context of other adjacent fibrochondrocytes in the full FIB-SEM volume. (D) Thickness maps from multiple FIB-SEM volumes at different mouse ages near the mineralization front were used to quantify and compare the extent of mineral packing in the calcified fibrocartilage zone of WT and *Hyp* enthesis at and below the mineralization front. Incomplete mineral tessellation is readily apparent in the *Hyp* fibrocartilage as compared to the WT fibrocartilage. Figure adapted from reference 48.

Recent investigations have contributed extensively to the structural biology understanding of entheses, and of calcifying enthesopathy in the *Hyp* mouse model. Using a similar X-ray and electron microscopy tomography approach combined with quantitative reconstructions and integrated with other conventional 2D electron microscopy methods from sections and tissue block faces, we observed at the Achilles tendon enthesis (Figure 6A) a similar delay (as in bone) in the developmental trajectory of fibrocartilage mineral tessellation in the *Hyp* mice as compared to WT mice.^49^ In both WT and *Hyp* aging mice (7 mo of age and above), Achilles tendon (midsubstance) likewise shows a crossfibrillar mineral tessellation pattern (an Achilles calcifying tendinopathy). This indicates that there is a shared mechanism occurring among different connective tissue and collagen-producing cell types (osteoblasts/osteocytes, fibrochondrocytes, and tenocytes) after an initial symmetry-breaking mineral nucleation barrier has been overcome to form mineral foci.^49^

Using X-ray tomography and 3D FIB-SEM imaging coupled with deep learning-assisted segmentation, the 3D organization of mineral was visualized at the mineralization front within the calcified fibrocartilage of the Achilles enthesis. In calcifying fibrocartilage of the WT enthesis (Figure 6B and C), a 3D crossfibrillar mineral tessellation pattern was observed similar to that of lamellar bone, but with mineral tesselles having greater variance in morphology and size.^49^ A similar distribution of tesselle aspect ratios was observed as in WT lamellar bone, where small mineralization foci within uncalcified fibrocartilage expanded to form space-filling mineral tesselles.^18^^,^^23^^,^^49^ Using watershed transform applied to complete tesselles, we computed the overall distribution of tesselle equatorial widths (the minimum Feret diameter) that generally aligns perpendicular to the long axes of both the tesselles and the collagen fibrils. This showed that a greater size variation of tesselles exists in fibrocartilage than in bone.^49^ Feret diameters of tesselles from under the mineralization front of WT lamellar bone had a unimodal distribution centered around 0.35 μm, whereas for a volume just under the mineralization front of enthesis fibrocartilage there was a large spread ranging from 0.30 to 1.3 μm. Boundaries between fibrocartilage mineral tesselles remained distinct, just as in bone, and extended deep into the calcified fibrocartilage zone that is directly adjacent to the subchondral calcaneal bone (with its intervening cement line/plane).

While WT mice display a characteristic trajectory of mineralization in the fibrocartilage from small mineral foci tens to hundreds of nanometers in size that grow preferentially along the long axis of collagen fibrils to form a regularly patterned, space-filling assembly with neighboring tesselles (Figure 6D, left two images), *Hyp* specimens lack such a mineralization trajectory and structure in their fibrocartilage^49^ (Figure 6D, right two images). Below the ill-defined *Hyp* mineralization front, mineralization is observed in 3D as smaller, more irregular, and isolated mineral volumes that do not abut against one another and instead have wide unmineralized spaces between them.^49^ As calculated from the analyzed fibrocartilage volumes, there is more than double the number of isolated mineral foci in *Hyp* (~7.6 per 10 μm^2^) compared to WT (~3.1 per 10 μm^2^) fibrocartilage that occupy the depth of any one 2D “window” when looking from the uncalcified fibrocartilage toward the calcified fibrocartilage zone at this scale. This is attributable to the persistence of arrested immature mineral foci over a large distance in the vicinity of the mineralization front. This contrasts with the WT fibrocartilage case where mineral foci are only observed immediately above the mineralization front, so their number in any one equivalent interface area is about half. In *Hyp* mouse fibrocartilage, as for *Hyp* lamellar bone, there is no clear mineralization front for an extended distance.^23^^,^^49^ OPN is abundant in both WT and *Hyp* fibrocartilage. Finally, considering the hallmark POLs found in *Hyp* bone, we explored whether *Hyp* fibrochondrocyte mineral lacunar volumes were increased compared to WT fibrocartilage, but this turned out not to be the case.^49^

For calcaneal bone, severe osteomalacia was obvious in *Hyp* mice by X-ray microscopy (submicron μCT). Some evidence of calcifying enthesophyte development was found in *Hyp* mice, although not at the same progression as has been reported in human patients. Also, protruding calcified structures (as enthesopathies) are not prominent in *Hyp* mice, although we occasionally observed some small, calcified protrusions in the *Hyp* mice, more common toward the plantar fascia surface.^49^^,^^100^ However, in terms of enthesopathy, major molecular changes in the fibrocartilage (including increased proteoglycan content) and gene expression profiles for the fibrochondrocytes (including increased alkaline phosphatase-positive cells and activity) occur at the Achilles enthesis in *Hyp* mice,^101^ and the reader is referred to works from the Liu lab for further details and discussion about these changes.^102^^,^^103^ Recent investigations have supported a significant overarching role for mechanical adaptation that may further exacerbate molecular and structural phenotypes, particularly in untreated adult patients.^104^

## Outlook for mapping pathologies onto the hierarchical organization of bone

The new insights into the pathobiology of XLH obtained using 3D multiscale microscopy opens a new way to consider classifying pathologic skeletal conditions. In the context of our contemporary understanding of the hierarchical structure of bone, the origin of rare monogenic diseases will always be mapped to the same level and structural unit. On the contrary, common multifactorial diseases can originate at many levels of bone hierarchical organization. For example, excessive nonenzymatic crosslinking of collagen, hypermineralization, excessive cortical porosity, or simply gracile phenotype may result in a fragility fracture. Moreover, multifactorial diseases can be aggravated or compensated across other structural levels. For example, tissue-level fragility of nonenzymatically crosslinked collagen can be aggravated by a gracile bone frame, but compensated for by superb neuromuscular coordination. This outlook offers finer granularity in distinguishing pathophysiologic pathways, explaining heterogeneity of patient populations, or matching a human disease with an animal model.

## Outlook for structural biology methods to study mineralized tissues

One of the biggest limitations in imaging science is the assumption that a small area of interest (at any length scale) can be “representative” of the tissue as a whole—a generalization that can become more implausible as volume size decreases. Whether examining bone trabecular morphometry at the microscale, canalicular density at the nanoscale, or localization of ionic calcium at the atomic scale, it is both natural and expected to see wide variation in healthy tissue structure with even a minor change (a few mms, μms or nms, respectively) in anatomical location. Therefore, when aiming to attribute structural abnormalities to a particular condition or disease, it is beneficial to perform repeated measurements across multiple specimens (defining a repeatable characteristic of structural decay) or to use workflows that precisely localize the anatomical region from which the imaging volume was extracted.^105^ In this review, we have described an array of multimodal 3D characterization techniques (X-ray, optical, and electron microscopies) that have been used to characterize WT and *Hyp* mouse bone, and XLH human bone to a limited degree. A detailed description of these and other techniques, along with a short description of their complementary forms of 3D spectroscopy, can be found below in the Addendum section. Although each technique can provide valuable information about a small number of hierarchical levels in bone’s expansive structure, no one technique can image the entire structural hierarchy of bone. The scientific community is making good progress in identifying and defining key correlative workflows that use multiple forms of 3D imaging on the same site within the same specimen to more completely examine structural hierarchy and add global context to local measurements.^7^^,^^9^^,^^106–110^ For instance, an ideal 3D characterization workflow may consist of:

1. Microscale characterization: Micro-CT of a whole bone in an animal model.
2. Localization of an area of interest from the 3D data and appropriate sectioning.
3. Mesoscale characterization: FIB-SEM tomography within the specified area of interest.
4. Extraction of a TEM lamella or atom probe specimen from the region immediately adjacent to the FIB-SEM volume.
5. Nanoscale characterization: Electron tomography of the TEM lamella or atom probe specimen.
6. Reshaping the TEM lamella into an atom probe specimen as necessary.
7. Atomic characterization: Atom probe tomography on the needle-shaped specimen.

*Note:* Imaging and cryo-preservation should be added where possible (see below).

3D imaging science is increasingly faced with the problem of big data—where image processing operations are the bottleneck of many 3D imaging workflows, and where analyses are unable to keep up with the relatively high rate of image acquisition. For future correlative workflows especially, it is important to develop accurate and automated reconstruction, registration, and segmentation algorithms to extract meaningful quantitative conclusions about the structural manifestation of osteomalacic diseases like XLH and HPP. Moving forward, we should aim to leverage modern computational infrastructure and recent progress in artificial intelligence to create high-throughput image processing suites that can be applied to correlative analyses of biological tissues. As 3D correlative methods are expensive and laborious, AI-aided navigation and sparse sampling will become widespread. Given the high costs and expertise required to acquire these image volumes, a recent publication has emphasized the importance of data sharing.^111^ Development of correlative imaging workflows (including for 3D volume reconstruction and segmentation) will standardize and enhance interpretability of these data within the bone and mineralized tissue community. This will not be a trivial feat given the challenges associated with imaging mineralized tissues as compared to soft tissues. With systems in place to appropriately credit the labs and facilities that generate the original data, data sharing will be crucial for the future of this field to increase accessibility of data and understanding of these techniques, and most importantly for advancing the knowledge of mineralized tissue diseases at this scale.^112^

**Figure 7:**
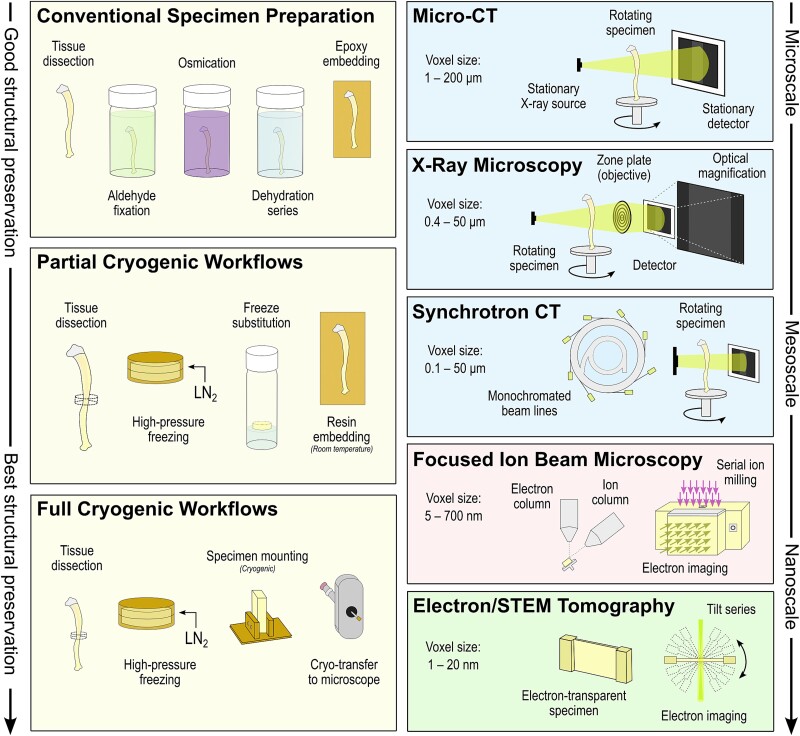
Overview of specimen preparation workflows and multiscale imaging techniques for structural investigation of mineralized tissues.

A final consideration is one of specimen preparation techniques and the precarious labile nature of both organic and inorganic phases (including precursor phases and mineral polymorphs) in bone tissue. Amidst the “resolution revolution”^113^^,^^114^ the use of traditional chemical specimen preparation methods (aldehyde-based fixatives, optional osmications, and acetone/ethanol dehydrations) should be reconsidered where possible in favor of (or in addition to) cryogenic sample preparation (Figure 7). By using high-pressure freezing, vitrification of water in thin hydrated samples (200 μm or less in thickness) can be used improve the preservation of delicate structural details in the tissue by physical immobilization rather than by chemical crosslinking—thereby preserving a tissue volume with little volumetric shrinkage.^106^ In partial cryogenic workflows (in instances where imaging or some manipulation of the sample must be done at room temperature), a high pressure-frozen specimen can subsequently be freeze-substituted by slowly warming the sample from cryogenic conditions in the presence of an organic solvent, helping maintain a structurally important fraction of “bound water” while purging the bulk/unbound water from the specimen. In partial cryogenic workflows, conventional resin embedding can then follow to create a final tissue structure that is better-preserved than a chemically fixed alternative.^115^ However, the best-known form of fine structural preservation is through full cryogenic workflows, where, after tissue dissection and high-pressure freezing of a specimen, imaging can also be conducted at cryogenic temperatures. Even with current technological developments, performing cryo-transfer of the specimen and volume electron microscopy at cryogenic temperature (without formation of damaging crystalline ice) are difficult processes to master.

To summarize, 3D multiscale imaging, and especially cryogenic 3D imaging that originates from the nanoscale or atomic scale, is essential to understand critical relationships between organic and inorganic tissue constituents in bone and cartilage. Similar to the early years of electron microscopy, 3D imaging alone does not tell the full story of disease progression, but serves as an excellent complement to genetic, biochemical, and other molecular assays—shaping a holistic understanding of tissue alterations in diseases as exemplified by the case of XLH (and osteomalacia in general) discussed here in this review.

## Addendum

### A summary of microscopy methods as used for structural features of bone

Based on the hierarchical ordering of components within skeletal tissue,^116^ research practices to characterize normal mineralized tissue structure, or structural abnormalities, benefits from a multiscale and/or correlative approach to observe the full scope of skeletal structure in health and disease. Depending on the structural level(s) where a given disease manifests itself, correlative approaches that utilize multiple techniques^9^ are often required to identify mineralization defects and any longitudinal adaptations/remodeling associated with a disease.

In general, 3D imaging volumes are generated either nondestructively through the acquisition of 2D projections in a rotation series and subsequent reconstruction into a stack of images (eg, most X-ray techniques, electron tomography), or the direct acquisition of an image stack through destructive means (eg, focused ion beam–scanning electron microscope [FIB-SEM] tomography, array tomography). Here, we provided a short summary of macroscale-to-atomic scale 3D imaging techniques available for use in clinical and pre-clinical research settings and for the study of skeletal disorders (Figure 7).

#### Clinical CT

In clinical CT systems, both the X-ray source and detector are rotated around the tissue of interest in tandem to generate a series of images based on the corresponding attenuation of X-rays.^117^ The images are then converted into a stack of images (producing a registered 3D volume) by iterative reconstruction techniques.^118^ The intensity of a given voxel is assigned a value according to the Hounsfield scale, where water is assigned a value of 0 H, and positive values denote tissue that absorbs more radiation than water, and negative values correspond to tissue/regions that absorb less radiation than water.^117^ The major benefit of clinical systems is the capability of necessarily imaging large volumes using a low dose of radiation. This means that longitudinal, in vivo imaging can be more easily applied to monitor the progression of a disease over time. A shortcoming of clinical CT compared to other forms of 3D imaging is a lack of resolution (with voxel sizes being on the order of 200 μm-1.5 mm).

#### μCT and submicron μCT (X-ray microscopy, XRM)

For higher-resolution 3D imaging using X-ray modalities, it is also possible to use μCT for imaging of mineralized tissues.^119^ μCT uses a stationary detector and polychromatic X-ray source, instead generating a series of 2D projections by rotating the specimen rather than the source-detector pair like in clinical CT. Similar to clinical CT datasets, the series of projections are reconstructed into an image stack for manipulation in 3D. An approach termed “geometric magnification” can be used to control the voxel size in μCT acquisition.^119^ Since the emitted X-ray beam is divergent (being a cone beam), the ratio of source-to-sample distance and sample-to-detector distance controls possible magnifications for a single projection. Moving the specimen closer to the source increases magnification, while moving the specimen closer to the detector decreases magnification. Further magnification can be achieved using XRM approaches that add an objective lens (consisting of a zone plate—a series of precise concentric slits that focus the X-rays onto a scintillator by diffraction) between the specimen and the detector. Select μCT systems can perform lower-resolution imaging in vivo for longitudinal monitoring of musculoskeletal disorders in mice or rats,^120^ but μCT and XRM techniques typically rely on tissue dissection and/or animal sacrifice due to the high dose of radiation exposure during relatively long scanning times. Although conventional μCT systems typically have a minimum voxel size of roughly 1-200 μm, the addition of advanced optics for optical magnification and geometric magnification approaches can push the minimum voxel size to as low as 100 nm. μCT and XRM systems are typically not able to scan as large a tissue volume as clinical CT systems, but they offer an excellent balance between resolution and specimen size.

#### Synchrotron radiation CT (SR-CT)

To accelerate the acquisition time of high-resolution 3D X-ray imaging, it is possible to use SR-CT for high-throughput analyses.^121^ To generate synchrotron radiation, electrons are emitted from a source in the center of a synchrotron facility, increasing the energy of electrons in the central booster ring of the synchrotron and guiding the electrons along a circular path. The electrons are then transferred to the outer storage ring, where magnetic fields are used to control the path of electrons and generate light. This light can then be harvested using beamline optics to extract an extremely intense beam of X-rays for CT. With such high-intensity X-rays, energy filtering devices can be applied to achieve a monochromatic beam to avoid radiation hardening effects and allow for quantitative image analysis during CT imaging. The high brilliance of the source allows for rapid scanning of skeletal tissue.^122^

The high brilliance of the source allows for rapid scanning of skeletal tissue (often <1 min for an entire specimen, depending on the exact type of measurement being conducted).^122^ Specimens for SR-CT are often much smaller than μCT specimens, and—especially for biological tissues—the intense X-ray beam can cause specimen damage during SR-CT acquisition,^123^ although protocols have demonstrated that complementary histochemical, immunohistochemical, and RNA/DNA analyses are still possible following irradiation.^124^

#### FIB-SEM and other volumetric SEM approaches

Although a destructive technique, FIB-SEM tomography uses a dual-beam system (with separate columns for ion and electron generation, for surface tissue layer ablation and imaging, respectively) to image microscale, mesoscale (between the microscale and nanoscale), and nanoscale features of mineralized tissue. Data is acquired using a serial milling/cutting approach,^125^^,^^126^ where the ion beam is used to remove (ablate) a thin layer of material from the surface of the tissue (usually on the order of 1-50 nm in thickness). The microscope stage is then rotated such that the newly exposed surface faces the electron column (or images can alternatively be acquired in the milling position with appropriate image rescaling depending on the angle between the ion and electron columns), and an image is taken using the appropriate electron detector. The process iterates across tens, hundreds, or thousands of milling and imaging steps to progressively image the volume slice-by-slice (much like serial sectioning in microtomy). Post-process image slice registration can then be applied on the image stack to create an aligned volume for 3D analysis. Fiducial markers are often added on both the top of the volume and the cross-sectional face to ensure that the ion beam mills in the correct location on successive slices and to aid in accurate image registration after the acquisition finishes, respectively. To protect nearby regions of the volume from unwanted damage during focused ion beam milling, a robust capping layer (typically 2-4 μm of carbon, platinum, or tungsten derived from a gaseous precursor) is deposited over the entire volume before 3D imaging begins.

In FIB applications (especially common in bone or mineralized specimens in general), focused ion beam milling can create curtaining artifacts, arising from inhomogeneities in the material where neighboring regions have an uneven sputtering rate. “Curtaining” artifacts in the specimen become more pronounced as the milling current increases. Some remedies for minimizing curtaining artifacts include the use of a “rocking polish” (changing the incident angle of ions between slices), or through post-processing image analysis.^127^ A major asset of FIB-SEM tomography is that other SEM techniques can be applied simultaneously during volumetric imaging. Recently, systems have become available that allow for slice-by-slice elemental mapping (by energy dispersive X-ray spectroscopy [EDS/EDX])^128^ or crystallographic orientation mapping (by electron backscatter diffraction [EBSD]).^129^ Although these complementary techniques greatly prolong the overall acquisition time, they offer additional potential for an in-depth quantitative assessment of tissue structure in healthy and pathologic tissues.

As an alternative to FIB-SEM, 3D tomography can also be conducted in the SEM using serial-block-face EM (SBF-EM) or array tomography. Serial-block-face EM uses an in-chamber diamond knife (microtome) to serially remove slices of material, acquiring a set of SEM micrographs from the tissue/block face between individual sections.^20^^,^^130^ In array tomography, ultramicrotomy is used outside of the electron microscope to generate a series of arrays/ribbons containing consecutive sections from the specimen (roughly 200 nm in thickness). Optionally, the array tomography sections can be stained post-sectioning to add contrast for imaging. The arrays are then loaded into the SEM, where they can be imaged in a sequentially ordered, section-by-section manner to create a 3D volume in an archivable fashion (where regions of interest can be revisited for additional characterization if necessary).^131^ Volumes generated by SBF-EM and array tomography typically have a disadvantageous anisotropic voxel size, meaning the resolution in the *Z*-direction of the image stack is less than the resolution in the *X*-direction and *Y*-direction due to the large section thickness relative to the pixel size in the imaging plane. FIB-SEM tomography, however, can be conducted in a way such that the slice thickness is set to match the pixel dimensions within the imaging mode, achieving a desirable isotropic voxel size in the final volume. Enhanced FIB-SEM instrumentation could have the capability of imaging full osteocytes and their perilacunar volume (including mineral and organic collagen matrix) in 3D and at very high resolution capable of capturing the metabolic profile of an osteocyte within a POL (by understanding changes in intracellular morphological features). However, it must be recognized that overcoming the aforementioned challenges of ion beam milling and electron imaging of mineralized tissue to generate such large volumes (as has been done for enhanced FIB-SEM of soft tissues) will not be trivial and has many challenges to overcome.^109^^,^^132^

#### Electron tomography

For 3D assessment of nanoscale structures in bone tissue, the transmission electron microscope (TEM) can also be used to conduct electron tomography. 3D volumes are generated from a single electron-transparent ROI, where a thin section is prepared through focused ion beam lift-out^133^ or microtomy to a final thickness of 200 nm or less. By acquiring a tilt series of the rectangular region (typically to a maximum tilt angle of ±60° in increments of 1°-3°), TEM or scanning transmission electron microscopy (STEM) images can be reconstructed into a 3D volume using iterative approaches (such as a simultaneous iterative reconstruction technique [SIRT] across 20-40 iterations) or applying a weighting factor in Fourier space (often the weighted back projection algorithm [WBP]).^134^ Following the reconstruction, the image stack should have an identical voxel size in both the *X*- and *Y*-dimensions. However, the *Z*-dimension of each voxel becomes slightly shortened after reconstruction, and an elongation factor can be appropriately calculated/applied using the range of tilt angles.^135^

Like in μCT, the tilt series is most effective when projections can be acquired across a 360° range of angles. In electron tomography, however, tilting beyond 60° is often difficult because the effective thickness of each tilted section increases with respect to the stationary incident electron beam (thereby reducing the transmitted signal) and spatial constraints in the microscope chamber. Additionally, for specimens mounted on mesh TEM grids, the grid bars can begin to obscure the image at high tilt angle. These “missing wedge” artifacts can be avoided using a specialized specimen holder and a technique called *on-axis electron tomography*,^136^^,^^137^ where a cylindrical specimen is shaped using focused ion beam preparation to permit a full 360° tilt series.

When the STEM modality is chosen for electron tomography, it is possible to generate compositional and crystallographic information in a voxel-by-voxel fashion. As the focused electron probe rasters across the ROI, an EDS/EDX detector for elemental detection can be used to capture characteristic X-rays from each point within the projection. As this is repeated for all projections within the tilt series (called STEM-EDS tomography), the volume can be reconstructed using the same techniques as above, this time preserving the chemical information within the specimen to generate 3D elemental maps.^138^^,^^139^ In a similar fashion, a 2D diffraction pattern can be generated at each pixel in a 2D projection to reconstruct a 3D map of crystallographic orientation (using a technique called 4D STEM tomography).^140^ For soft materials, the accumulation of damage from the electron beam can be appreciable during basic electron tomography, and the specimen is further damaged if additional beam exposure is needed for these complementary techniques.

In instances of single-particle cryo-EM (where thousands of replicas of the same particle/protein with different orientations can be distributed across a TEM grid for similarity binning and statistical analysis), a 3D volume can be generated without the use of a tilt series.^141^ Following dispersion of proteins/particles onto the grid, the grid can be plunge frozen to preserve the fine structural details within each instance of the protein/complex. Automated workflows exist that can then capture tens or hundreds of thousands of 2D images of the protein/peptide in the TEM (in random configurations, capturing the particle in nearly every possible orientation) and reconstruct the particle with a resolution of 2-4 Å depending on the microscope optics, detector and number of images acquired.

#### Atom probe tomography (APT)

To spatially examine preferential localization of ionic species within the bone, APT is one of the highest-resolution 3D imaging capabilities available for mineralized tissues and other materials.^142^ In APT, a focused ion beam or electropolishing apparatus can be used to produce a needle-shaped specimen with a tip radius of <100 nm. By pulsing a laser onto the tip of a specimen and placing a counter-electrode near the specimen tip, local field evaporation can occur as individual ions are accelerated to a position-sensitive detector (recording the time and location of impact). A mass spectrum is produced as field evaporation continues, where, based on the mass-to-charge ratio of the particular ion or ionic complex, the initial position of the atoms can be estimated. Figure 7 schematically highlights major features of sample preparation workflows and imaging.

## Data Availability

The authors confirm that the data supporting the findings of this study are available within the article.
